# A process evaluation of the Mind Your Back trial examining psychologically informed physical treatments for chronic low back pain

**DOI:** 10.1186/s12998-021-00389-y

**Published:** 2021-08-17

**Authors:** M. John Petrozzi, Grace Spencer, Martin G. Mackey

**Affiliations:** 1 Faculty of Medicine and Health, The University of Sydney, Level 7, Susan Wakil Health Building D18, Sydney, NSW 2006 Australia; 2 Faculty of Health, Education, Medicine and Social Care, Anglia Ruskin University, Cambridge, UK

**Keywords:** Chronic non-specific LBP, MoodGYM, Psychologically informed physical therapy, Qualitative process evaluation

## Abstract

**Background:**

In chronic conditions, such as back pain, the use of interventions that address physical, social and psychological aspects within a biopsychosocial framework are encouraged, however, applying this holistic multimodal approach in physical therapy practice (i.e., chiropractic and physiotherapy) is challenging. To explore the problem of delivering a biopsychosocially informed package of care in physical therapy practice a recent randomised control trial (RCT) called ‘Mind Your Back’ was conducted to evaluate the effectiveness of a combined physical and internet-delivered psychological intervention (psychologically informed physical treatments) compared to standard treatment for improving disability and self-efficacy in people with chronic LBP. The results of the trial indicated no difference between the two intervention groups. Although high-quality RCTs are considered gold standard for effectiveness of interventions, qualitative research methods embedded within a process evaluation framework are also used to reveal other issues and important information that help to explain clinical trial results, and to further the field of digital health interventions research. Therefore, within a process evaluation framework, the aim is to explore participants experiences of the interventions received throughout the Mind Your Back trial which led to a null result.

**Methods:**

In-line with recommendations for a process evaluation this study used in-depth interviews and qualitative thematic analysis with participants of both arms of the trial 5–6 months after study completion. Semi-structured telephone interviews were conducted with twenty-five participants to explore their experiences of taking part in the Mind Your Back trial. Interviews were conducted in November 2017, transcribed verbatim and data analysed thematically.

**Results:**

Two main themes were identified: (1) Personalised support and therapeutic alliance are important, and (2) MoodGYM lacked relevant, personalised and tailored support.

**Conclusion:**

It is important to deliver tailored digital health supports that is personalised and fosters a therapeutic alliance.

**Supplementary Information:**

The online version contains supplementary material available at 10.1186/s12998-021-00389-y.

## Introduction

In chronic conditions, such as back pain, complex interventions that use a biopsychosocial framework are encouraged [1–5]. However, applying this holistic multimodal approach in physical therapy practice is challenging and has not been extensively investigated to guide clinicians. Consequently, a multisite randomised controlled clinical trial, ‘Mind Your Back’, was conducted recently to evaluate the effectiveness of a psychologically informed model of physical therapy care for 108 people with chronic non-specific LBP at medium-risk of ongoing disability [6, 7]. Although, randomised controlled trials are considered gold standard for effectiveness of interventions, qualitative research methods are also recommended to reveal relevant information to help explain the processes involved in implementing a complex health intervention [8]. Process evaluations within clinical trials are used to explore the implementation of a complex health interventions and examine participant views within the settings of the trial to aid the interpretation of the trial results [9].

Process evaluation can be used to discriminate between faulty interventions and poorly delivered interventions [10]. According to Oakley et al., studies using a process evaluation are especially important in multisite clinical trials, where the “same” intervention has been implemented, as it is likely to have been received by participants in different ways [11]. The key recommendations for planning a process evaluation include a clear description of the study aims linked with the trial objectives, selection of an appropriate qualitative methodology to inform data collection and interpretation, and use of expert research personnel with experience in qualitative methodology, and human research ethical approval [8].

In line with recommendations for undertaking a process evaluation within a clinical trial, this paper applied these approaches to evaluating the Mind Your Back trial. The Mind Your Back trial included two groups of 54 individuals. Group 1 received multimodal physical treatments by a chiropractor or physiotherapist and group 2 received the same physical treatments combined with an internet-delivered psychosocial program called MoodGYM. The primary outcomes of the trial were low back related disability and self-efficacy which were measured at baseline, 8-weeks, 6- and 12-months. The results of the trial revealed no statistically significant difference in disability between the two groups at any of the follow-up timepoints (SMD − 0.06, 95% CI − 0.45 to 0.31) or self-efficacy (SMD 0.06, 95% CI − 0.31 to 0.45). Although, this was a high-quality randomised clinical trial informed by clinical practice guidelines, the null results require further investigation to inform clinicians on the use of psychologically informed physical therapy. To that end, a process evaluation was undertaken with trial participants to help understand their experiences of the intervention and crucially, to help explain the null results of the trial. The findings of this process evaluation can also help to further the field of digital health interventions research.

## Methods

### Study design

In-line with recommendations for the design of a process evaluation the objective of this study was aligned with the aims of the clinical trial [8, 11]. Also, as recommended by guidance [8, 11], this process evaluation was conducted with participants of both arms of the trial 5–6 months after study completion. The data was collected using in-depth interviews as this is considered an appropriate qualitative method for exploring participants’ experiences of interventions received throughout a clinical trial [8, 11]. Furthermore, as recommended in process evaluation guidance, this study is supported by experienced qualitative personnel and has ethical approval [8, 11].

### Participants

A convenience sampling strategy was used to recruit trial participants for this qualitative process evaluation as some individuals were known to the interviewer throughout the trial. That is, participants that had been treated by the interviewer in a professional capacity during the Mind Your Back trial were not invited for interview. Therefore, of the 108 trial participants, 61 were invited to take part in the process evaluation, of which a total of 32 participants volunteered to be interviewed in November 2017. Interviewees were contacted sequentially from the volunteer’s list and were recruited based on their availability and consent to participate in the interview. The interview process was discontinued after 25 participant interviews based on the principles of data saturation [12–17] as respondents were consistently repeating similar views, statements, phrases and no new ideas were shared by the interviewees. The remaining 7 volunteers were contacted and informed that they would not be participating in an interview.

### Data management

Participants were assigned a pseudonym for anonymity and reporting purposes. Data was securely stored on the University of Sydney Research Data Store and will be kept for 15 years after the completion of the study.

### Interviews

Semi-structured telephone interviews were conducted. Telephone interviews were deemed most feasible and convenient because participants were located in both NSW and Victoria. A flexible interview guide (Table 1) was designed to capture participants’ response to interventions provided in the Mind Your Back Trial centred on the following discussion areas:

- Expectations about participating in the trial
- Experiences of participating in the trial and perceptions of the benefits and difficulties of interventions
- Overall perceived effects of the intervention

**Table 1:** Interview guide questions

*Pre-trial expectations*
Can you tell me a few reasons why you chose to take part in the trial?
How did you expect that the internet-delivered mood program would help you?
Did you think that a computer program could help you manage your mood better?
*Usual care experience*
Can you tell me what you thought of the chiropractic/physiotherapy you received?
Was the treatment enough on its own to manage your pain? Or was something missing?
What else would you like to have received from your practitioner?
*Intervention experience*
If you were in the MoodGYM group: How did you feel when you first heard that you would be using MoodGYM in addition to chiro/physio in the trial?
After you started using MoodGYM each week, how did you feel about it?
Can you tell me about how you managed to get through the modules?
As you went through each module, what emotions and thoughts came up for you?
What did you think about the modules presented in MoodGYM?
What benefits did you experience from using MoodGYM?
*Intervention relevance*
What relevance did MoodGYM provide for you?
Would you recommend MoodGYM to someone dealing with emotional distress like anxiety or depression as a result of chronic back pain?
*Intervention improvements*
What would you have changed about the MoodGYM to make it more relevant to your situation?
What would make your experience in the trial better?
*Overall perceived effects*
How did your life change as a result of participating in the trial?
Is there anything else you would like to tell me about your involvement in the trial before we wrap things up?

The interview guide included open ended questions enabling participants to freely discuss important aspects of their condition and how it related to the trial, as well as more specific questions regarding their experience of the trial interventions to provide insight, or explanation for the null results. The discussion guide was piloted with a small group of participants who were not involved in the interviews. Minor adjustments were then made to enhance clarity and fluency. Interviews lasted between 20 and 45 min, mean 35 min, and were digitally recorded and transcribed verbatim by a professional transcription service. Brief summary notes were made after each interview in relation to the broad discussion points and any extraneous factors relating to interviewer/interviewee rapport and reactivity. The latter were reflected upon during analysis and considered in the final discussion.

### Data analysis

In keeping with the qualitative methodology, a thematic analysis [18, 19] was chosen over other qualitive methods (e.g., content analysis) as it is an interpretive process, allowing a systematic identification of patterns within data to emerge. The thematic analysis was undertaken using a multi-stage process. First, transcripts were read and re-read to grasp an overall impression of the data. Second, initial codes were used to label features of the data that might be relevant to answering the research question. Third, descriptive codes were added to the transcripts to reflect participants’ meanings and this process of coding identified several salient ideas important to participants such as ‘the fluctuating nature of low back pain’, ‘coping strategies utilised to manage low back pain prior to the trial’, and ‘positive and negative experiences of the trial interventions’. Fourth, codes were discussed and scrutinised by three researchers to identify significant broader patterns of meaning and grouped into categories. Fifth, the research team further discussed these categories and generated some potential themes. Sixth, potential themes were checked against the transcripts to determine that they accurately reflected the data, and answered the research question. Finally, the team reached agreement by comprehensively and rigorously discussing the findings and potential themes, allowing for different interpretations of the data that best strengthened the analysis, and decided on the final themes. The reporting of the data collected follows the Consolidated Criteria for Reporting Qualitative research (COREQ) for reliable reporting and reproducibility of findings [20] (Additional file 1).

### Ethics

Ethics approval was obtained from the University of Sydney Human Research Ethics Committee (2014/997). Participants were provided with a Participant Information Sheet that outlined the aims and personal considerations of taking part in the qualitative study. All participants provided signed consent prior to taking part in the study. At the start of each interview, participants were reminded that they were not obliged to answer the questions asked and that withdrawal from the interview was possible at any time without giving reason and that their personal information would be securely stored on the University of Sydney Research Data Store servers.

## Results

The interviewed participants (N = 25) shared similar demographic characteristics to the 108 individuals in the Mind Your Back trial. That is, the interviewees consisted of 12 women and 13 men, with an age range from 29 to 76, mean 53 (SD = 13) who had lived with chronic low back pain for an average of 4.3 years. Furthermore, as in the Mind Your back trial which consisted of two evenly grouped intervention arms, interviews were conducted with fourteen individuals from group 1 (physical treatments only), and eleven from group 2 (combined MoodGYM and physical treatments).

Participant’s recount of their response to intervention prior to and during the Mind Your Back trial are grouped under the two major themes of (1) personalised support and therapeutic alliance are important, and (2) MoodGYM lacked relevant, personalised and tailored support for the management of chronic low back pain. Intervention groups are identified as G1 (physical treatments only) and G2 (combined MoodGYM and physical treatments).

### Personalised support and therapeutic alliance are important

Participants recounted different strategies they used, prior to the trial, to help manage their pain including informal strategies and those led by health professionals. Professional-led strategies prior to the trial included seeking advice and treatment from a GP, chiropractor or physiotherapist. Informal strategies included discussions with family and friends, as well as self-medicating with non-prescribed analgesia. The seeking of both formal and informal treatments seems to show the need to better personalise management of back pain as on average participants had lived with their back pain for over 4 years.

Prior to the trial support from GPs included prescriptions for strong analgesia and/or referral to other health professionals such a physiotherapist. Using prescribed medication such as opioid-based analgesia was reported positively to offer some relief from pain and allowed patients to regain some level of ‘normality’ through being able to participate in activities such as walking or getting to sleep at night.

After the seven years of having the constant pain, I went to my doctor and he said, "I want to try you on Tramadol," and I was a bit nervous about it at first, but it seems to be the only thing that really did kick in eventually and help. (Jenny, age: 48, G2)

I have to take painkillers before I go (for a walk) and painkillers again when I get back. It’s the only thing that seems to really help me. (Ivy, age: 55, G1)

Despite the positive short-term effects of analgesia, others indicated a preference to seek ‘hands-on’ treatment from a physiotherapist or chiropractor, which included massage, spinal manipulation, stretches and exercises. The familiarity with physical modalities seemed to motivate some participants to volunteer for the Mind Your Back trial and preference for personalised support. The responses of participants indicated that personalised care from a health professional that tailored treatment to their individual needs was preferred to taking pain control medication for their condition.

After checking on my response to the exercise I was then given a very specifically focused massage with some gentle manipulation type movements. (Mike, age: 76, G2)

I'm not into medication. That's just a band aid for me, so I'd rather avoid it. I’d rather go to the chiropractor or physio or something like that. (Carly, age: 49, G1)

Feedback from participants suggested that chiropractic treatment and physiotherapy offered longer-term benefits than medication and that adhering to regular or ‘maintenance’ exercises helped to improve their pain.

I go [to the chiropractor] for maintenance because my flexibility and my ability to keep moving improved with treatment. (Greg, age: 65, G2)

Positive engagement in clinical encounters by participants appeared to be closely tied to the perceived qualities of the practitioner. Such qualities included being perceived to be trustworthy, personable, friendly, positive, a good listener, approachable, caring and helpful. Participants valued practitioners who showed a genuine interest in their condition and demonstrated understanding of their personal circumstances and difficulties.

(The) practitioner … understands my situation, my individual situation, and (was) motivated to help me through that. It's very good having a trust relationship with your practitioner. Pretty important that I get treatment that's specifically for me, his manual therapy was targeted. The treatment and the whole interaction was very specific for my problem and personalised, to me as a person. (Diana, age: 43, G1)

I just found that he understood what I was going through as well. If you feel that someone can listen to you, and they feel that they can help you, then it's nice to know that there's someone there that you can turn to if you're in that pain, and you feel that someone is actually listening to you and understanding it. (Jenny, age: 48, G2)

The first thing about the practitioner was the relational ability. It's like he did this wonderful connection with me as a person. And then stayed relational all the way through. (Mike, age: 76, G2)

Personalised tailored advice and education about various exercises provided by the practitioner was also viewed positively. Perhaps exercise prescription was seen to enhance a tailored approach to their back pain care and engender a therapeutic alliance. Significantly, conversations with an encouraging practitioner helped individuals to better ‘come to terms’ with, adjust to and accept their pain and uncertain future.

Yeah, definitely, he encouraged me to stay active even if it was feeling painful, but just not to push it too far, but still do things. […] Yeah, definitely, his encouragement helped me a lot. (Jerry, age: 53, G1)

Well the treatment did help me, also his advice was really good. You know the guy that I saw finally pushed me to get a standing desk rather than sitting down at work all day. (Henry, age: 48, G1)

The guy treating me talked about this too, which helped me come to terms with it. I've got more acceptance to it, and I've got used to it. (Helen, age: 65, G2)

Despite these positive reports, participants expressed a dissatisfaction with treatment experienced prior to the trial and talked about the unhelpfulness of both the therapist and the therapies used. However, during these discussions, participants also echoed the importance of being understood and taken seriously by health professionals.

Oh, I suppose there would have been about eight or ten clients in his big treatment room, and he was just going from one to the other, and you didn't have the same one on one interaction, and so it didn't have the same encouragement, and, from where I was at the time, I just felt I was one of the numbers. (Kevin, age: 70, G1)

(I’d) often visit the GP, they get so sick of you. (Carly, age: 49, G1)

Other participants who maintained a more positive outlook about their pain expressed the idea that managing their pain was within their personal control. As a result, rather than seeking professional help, many described their pain management as being their own responsibility.

Long term, well, it's really me taking responsibility for myself, not relying on other people to fix me [… I] just try to remind myself of what I need to do such as the pacing, and looking after myself, from a nutrition point of view, being mindful, and trying to be positive. (Carly, age: 49, G1)

[The next time I’m in pain] in the future, I would try and self-manage first. (Betty, age: 51, G2)

### MoodGYM lacked relevant, personalised and tailored support

Participants expressed mixed views about the effectiveness and relevance of the digital health MoodGYM program used in the Mind Your Back trial (Table 2 describes the MoodGYM program modules). Positive experiences highlighted by several participants included that the program content provided the emotional support they were seeking, as well as provided access to information and reassurances about their emotions and (low) mood. Yet many others expressed negative experiences of MoodGYM, questioning its relevance to their LBP experience. Specifically, that the program content failed to address their specific physical needs (i.e., advice for managing the symptoms of back pain) and psychological needs (i.e., support for the emotional and social consequences of ongoing back pain), and the delivery method was reported as being impersonal (i.e., no human interaction).

**Table 2:** Description of the interventions used in the trial

Intervention	Description
MoodGYM	*Setting* Participants completed the MoodGYM program individually on their own personal computer at home
	*Purpose* To provide psychological support via an internet-delivered cognitive behavioural approach
	*Materials* Participants were directed to the MoodGYM website www.moodgym.com.au and asked to complete the five weekly modules. The modules explored thoughts, feelings, stressors and relationships that may contribute to psychosocial distress Module 1 Feelings: Why you feel the way you do Module 2 Thoughts: Changing the way we think Module 3 Unwarping: Changing warped thoughts Module 4 De-stressing: Knowing what makes you upset Module 5 Relationships: Relationships and how they work out
	*Procedures* One MoodGYM module was completed weekly. Fidelity was checked with a weekly telephone call by a research assistant. In circumstances that a participant reported not having completed a weekly MoodGYM module, a further phone call was made a few days later to ensure the module was completed. No additional counselling or psychological treatment advice was provided with these reminder telephone calls. The program was a self-led digital health technology with no contact with a health practitioner
Multimodal physical treatments	*Setting* Participants attended a private chiropractic or physiotherapy clinic. Physical treatments were provided by a registered chiropractor or physiotherapist with over 5 years of clinical experience. These practitioners were screened and inducted into the trial several months before the trial commenced
	*Purpose* To provide practitioner-led multimodal physical treatments focused on reducing back pain and help participants to better self-manage their condition
	*Materials* All participants received a pragmatic course of multimodal physical treatments, e.g., manual therapy (spinal manipulation or mobilisation and/or soft tissue massage) combined with reassurance, advice, education and general exercises. Reassurance that back pain would not worsen. Advice about symptom management and encouragement to remain active and avoid bed-rest. Education on activity pacing, lifting advice, computer ergonomic use and general injury prevention principles. Supportive exercises included general physical conditioning or home-based stretching and strengthening exercises relevant to the patient’s level of impairment and function. Treatment modalities that are not endorsed by clinical practice guidelines for the treatment of non-specific LBP were not offered to participants (e.g., therapeutic ultrasound, transcutaneous electrical nerve simulation, heat therapy, etc.). The selection of physical treatments was determined by the practitioner according to the presenting needs of the participant and according to clinical judgment
	*Procedures* Participants received up to 12 treatments over a period of 8 weeks. The practitioner may have elected to use fewer treatments in cases where significant improvement was observed or if adverse events that warranted stopping care were experienced. Fidelity and treatment adherence were recorded by the treating practitioner at each visit

NB: Intervention description follows the TIDieR (Template for Intervention Description and Replication) Checklist

Positively, the program seemed to help participants with the idea that feeling emotionally down was a normal reaction to chronic pain. The resulting reassurance enabled some to recognise their feelings of depression.

It made me realise that it is quite normal to feel certain emotions when you're not feeling 100%. Yeah, so it was good. It just reassured me that I wasn't losing my mind, basically. (Jenny, age: 48, G2)

I think [MoodGYM] was good to get your head around how you feel. I never took any notice of [my emotions]. I probably was depressed, and I didn't realise it. (Helen, age: 65, G2)

The resulting awareness and reassurance about how they felt enabled some participants to take positive steps towards adjusting their daily activities. For example, Emma seemed surprised by the support and advice that she received from MoodGYM, which helped her better understand her thoughts and emotions. This ultimately enabled her to better navigate her day-to-day life and feel in control of her mental health.

It was, actually, really brilliant […] there was some seriously great takeaways […], like, what you think about is what you feel, is probably the biggest one. [...] I think probably the fact that, how little I knew about how to control my mental health. It's quite a revelation, learning that stuff, and then applying it in my day-to-day life. (Emma, age: 35, G2)

Similarly, Helen’s account reinforced the idea that participants could be in control of their thoughts and moods, even in the context of ongoing pain. Managing moods and being positive appeared to have important implications for their social relationships.

It just brought it to the light to me, I have control on my thoughts and moods, even if the pain was there. I think it's made me more positive. Easy to get on with a bit more. People around me don't have to cope with my moods. I think it has done a lot of good for me. (Helen, age: 65, G2)

Despite positive accounts, participants reported negative experiences with MoodGYM—often citing the program’s lack of personalised (back pain) treatment and support. During these discussions, participants described the lack of back-pain-specific content, which made it difficult for participants to relate to the presented case material. Of importance was the idea that MoodGYM did not seem to address their primary concern, namely managing back pain.

Maybe they could be specific about someone who is actually going through [back] pain… being very specific about it. (Jenny, age: 48, G2)

In terms of the relationship [of MoodGYM contents] to the back pain, it wasn’t clear. (Betty, age: 51, G2)

Participants described difficulty relating to the apparent (dominant) focus on depression and without addressing the experience of living with chronic back pain. Karen, for example, described herself as not being depressed about her back pain and thus did not ‘fit’ into the program’s categories—a feeling echoed by other participants:

I found it [MoodGYM] okay, except that I didn't feel that I fitted into a lot of the categories, because even though I'm in pain a lot, I'm not depressed about the pain. (Karen, age: 66, G2)

It probably didn't add any value because I wasn't feeling too depressed about the pain when I did Moodgym. (Barry, age: 51, G 2)

Lack of personalised (back pain) treatment and the focus on depression led participants to suggest that they would not recommend MoodGYM to others with chronic back pain. When asked about the sorts of things they would like to see in an internet-delivered back pain program, Fred, expected the program to give him a visual representation of the causes of their pain. Betty and Alex wanted a description explaining the connection and impacts on mental health from chronic back pain.

A computer program, it would have to be quite graphic. It would have to explain the causes of the pain. And almost educate me… the way pain manifests itself. What it does to the brain. What it does to the chemical composition in your body. All that sort of stuff. (Fred, age: 68, G1)

I guess linking pain to mood. Like, the impact pain has on you, how that changes so you can recognise yourself [in the program], why a change in mood. Maybe how to manage it, if it hits. You know, different strategies to be tried [when experiencing episodes of back pain]. (Betty, age: 51, G2)

Maybe the program, if they would have been a bit more direct about that link [between back pain and low mood], it might have helped a bit quicker. (Alex, age: 29, G1)

MoodGYM lacked personal human connection and interactivity due to its computer delivered format. The importance of having personal human interactions with health professionals came through strongly, as participants cited the importance of building a relationship with practitioners when discussing their experiences of MoodGYM. The impersonal aspects of MoodGYM were further compounded by the reported technical difficulties of internet-delivered programs.

In terms of, I guess an online sort of thing, I guess personally, a personal interaction is probably more influential for myself than say going online and looking at a computer… because, you can't really ask it questions and clarification, if you know what I mean. Whereas if I'm talking to a chiro or a physio I can say, look, okay, am I doing this right. (Barry, age: 51, G2)

I'm not that tech savvy. I don't even know when I started, I think I did it twice, and then I don't even know whether I saved it, or what I did, and ... Yeah. Being older generation, I didn't grow up with all this technology. I’m not interested in anything on a computer. I believe in face to face. Well, I like face to face stuff. I don't like, sort of, self-directed learning, or guidance from a computer screen. (Carly, age: 49, G1)

The need for relevant support, that reflects the physical, emotional and social experiences of people with ongoing back pain, was not met by MoodGYM. The expectation and preference for a human interaction with an understanding and personable support mechanism, that provided tailored advice and treatment, was not provided by MoodGYM.

## Discussion

The aim of this study was to explore participants experiences of the interventions throughout the Mind Your Back trial using a process evaluation. Qualitative methods were selected to evaluate the trial and enable participants to discuss the factors that affected their experience of the intervention and help explain the trial outcomes. This process evaluation found two important themes (1) Personalised support and therapeutic alliance are an important part of back pain interventions, and (2) MoodGYM lacked relevant, personalised and tailored back pain support (Table 3). The first theme finding echoed the conclusion of a recent systematic review [21], that found strong therapeutic alliance was underpinned by health practitioner qualities of being understanding, trustworthy, approachable, and relational. The results indicated that a practitioner with these positive qualities was able to understand the lived experience of their patient and better form a therapeutic alliance. Findings highlighted the importance of the role the practitioner took. Together with the practitioner possessing a positive attitude, the building of an effective therapeutic alliance with the patient seemed especially important to the patients for understanding their everyday lived experiences. Furthermore, evidence suggests that, in the past, there has been a disconnect between the delivery of treatment interventions and the lived experience of people receiving interventions for chronic LBP [21–29]. The present study demonstrates the importance of providing patients with an opportunity to talk about their back pain in-depth, receive tailored advice and interventions that are sensitive to the lived experiences of chronic LBP. A recent study highlighted that efforts to optimise clinician–patient communication improved perceived treatment outcomes in primary care consultations [30]. Another study investigating the effect of the examination process (history taking and physical examination) on low back pain and disability found that the personalised evaluation process produced improvements in the therapeutic effect of back pain interventions [31]. Thus, it is important to tailor communication, advice and treatment that is congruent to the patients experience of physical and emotional distress and disrupted social life due to chronic low back pain.

**Table 3:** Process evaluation themes

Theme 1	Personalise support and therapeutic alliance are an important part of back pain interventions
Theme 2	MoodGym lacked relevant, personalised and tailored back pain support

Findings from the present study also suggest individual participants varied in their preference for the settings and interventions used to manage their condition. For example, some participants recounted preferences for face-to-face care with a health professional prior to the trial as it provided a therapeutic forum to discuss their personal experience and expectations, and receive tailored treatment, advice and encouragement that facilitated an adjustment to living with chronic LBP. Encouragement and advice communicated in a relational manner allowed participants to feel understood and more willing to accept that back pain was part of their life. Important aspects of this face-to-face therapeutic alliance include the affective bond and agreement of patient tasks and treatment goals between a patient and their practitioner [32]. Some participants described care-seeking prior to the trial and preferred treatments and strategies that offered immediate and targeted pain relief, such as prescribed pain medication and referral for enhanced diagnostic and specialist care, suggesting that pain and disability severity were motivating factors for seeking medical care [33]. Still others sought ‘hands-on’ care from chiropractors or physiotherapists that focused on providing personalised and tailored pain relief, management and prevention strategies through the use of physical treatments and exercise [33]. In contrast, other participants preferred self-help strategies such as self-medication with over-the-counter analgesia, performing home-based exercise and stretching, modifying their physical activity and avoiding triggers for their LBP. In summary, offering patients a supportive therapeutic alliance that encourages interaction in a safe and non-judgemental manner has the potential to help enhance the therapeutic alliance.

Another important finding of this study was that the use of a digital health technology, such as MoodGYM, was perceived as impersonal and did not provide back pain relief, nor did it assist patients dealing with any psychosocial dimensions of their chronic LBP. The content and delivery of this digital health intervention was generally perceived by trial participants as irrelevant, impersonal, and not tailored to their individual experience of chronic back pain. Some participants were more satisfied with the personal interactions they had with their treating health professional throughout the Mind Your Back trial than with interactions with MoodGYM program. Some participants’ preference for interaction with a healthcare professional seemed to impact their perspectives on the intervention throughout the trial. For example, their responses seem to indicate that MoodGYM was less preferred than the face-to-face consultations with health practitioners which provided a supportive environment that allowed them to share their experiences of living with LBP, communicate their needs for pain relief support and receive tailored advice and personalised management strategies. While MoodGYM is an internet-delivered program aimed to address emotional concerns, participants’ accounts suggest they expected the program to provide additional advice to manage the physical aspects of back pain.

These insights are important to both the design and delivery of face-to-face as well as internet-delivered support mechanisms for chronic low back pain as they are key to patients perceived relevancy for the intervention. An ability to relate and connect with important others (e.g., heath providers, peers, family) has been identified as an important factor that leads to enhanced health outcomes [34–37]. The need for attending to the patient’s narratives and experiences is seen as another way of improving therapeutic alliances [38]. These important relational aspect of collaboration for managing LBP did not feature in the MoodGYM program and may be an important reason why the clinical trial found no additional improvement in the pain and disability for those participants. Indeed, some participants described a need to discuss their personal circumstances while using MoodGYM; however, this was not possible due to the non-interactive nature of the online intervention. Some participants were critical of the content of the program; they perceived it was not well tailored to their lived experiences and did not reflect the physical and psychosocial disruption and complex adjustment they felt in living with chronic LBP. These findings also reflect the views of Multiple Sclerosis patients who took part in a trial using MoodGYM [39] and highlighted that digital health interventions need to provide a personalised and relational experience, that develops a therapeutic alliance, grounded in the real-world experiences of people with a chronic health condition [40]. The importance of therapeutic alliance to digital health technologies has been revealed by this study, and future studies need to focus on how best to integrate ways of enhancing the patients need for being listened to, understood and offered tailored interventions that can help them to accept and adjust to life with ongoing back pain.

In light of the findings in the present study, it is recommended that digital health technologies for low back pain need to offer tailored advice and personal support that facilitates management of this chronic condition, while capitalising on the convenience of an online environment. Recent studies for insomnia have trialed the use of avatars, in place of health professionals, in a fully-automated self-help program driven by an algorithm that provided tailored feedback and advice for insomnia [41]. The design of an internet-delivered program for chronic LBP could be enhanced by featuring avatars and characters that reflect the real-world physical and psychological disruptions experienced by people with chronic low back pain and that account for various stages of their adjustment. Furthermore, digital health technologies could enhance a sense of personal connection for users by providing a communication forum (e.g. chat rooms) with other users living with chronic LBP, and health professionals for advice and support. Aspects of this type of blended care, through brief telephone support with a health professional while completing an online intervention, have been used with success by internet-delivered cognitive behavioural therapy programs for depression [42]. It was found that users were motivated by the intermittent human contact to persist with the online intervention as they experienced a sense of belonging, relatedness and connectedness with the internet-delivered intervention. Designed in this way, a digital technology for people with chronic LBP can provide personalised best-practice management within a convenient digital environment and through supportive connections with others.

Despite these insights, there are some limitations to the study and its findings. Participants’ recall of trial experiences may have been affected by the 4–5 month period between the end of the trial and the interview. Participants were drawn from a relatively small sample of participants (N = 25) from the Mind Your Back trial (N = 108). Participants of the trial were at medium-risk of ongoing disability and findings may not be relevant for those with low or high risk of ongoing disability. The interview potentially may have represented a medical encounter [43] and may have elicited a partial account of participants’ experiences as it may have affected their willingness to express their full views. However, low participant reactivity was noted throughout the interviews which was supported by a flexible interview guide with open-ended questions and good rapport with the interviewer. Through the use of a reflexive journal, capturing the interviewer’s reactivity [44], it was noted that there was little influence on the participants ability to express their views in an uninhibited and natural manner. While participants’ accounts provided contrasting perspectives, consistency of responses across interviews was evident and suggested common experiences and challenges were faced by participants. Indeed, data drawn from interviews suggests that participants felt comfortable and willing to share their experiences. A further limitation of this study was that only one data coder was used to analyse interview transcripts. It has previously been stated that different conclusions can be derived from the same information depending on the personal characteristics of the researcher [45]. However, although one coder was used, greater rigour to the coding and interpretation was provided through in-depth discussion by the three-person research team to identify the core themes from the interviews and different interpretations of the data. A broader limitation of this study is that the process evaluation was not embedded in the design of the clinical trial as recommended by process evaluation planning recommendations [8, 11], but rather designed after the initiation of the trial. However, potentially the prospective design of the process evaluation may have produced a richer view of participants experiences as the null result outcomes of the trial were already known at the time of constructing the research project.

Findings from this process evaluation of the Mind Your Back trial highlight the importance of developing digital health interventions that provide tailored personalised support and relevant management strategies for people with chronic LBP. Facilitating a strong therapeutic alliance between patient and digital health interventions is a challenge due to the relatively impersonal and non-relational nature of digital interactions. However, by integrating opportunities for consultations with a health professional (e.g., face-to-face, video or chat) along with a highly relevant back-pain-specific internet-delivered program that develops a therapeutic alliance may help to enhance the delivery and relevancy of online interventions.

## Conclusion

It is important to deliver tailored digital health supports that are personalised and foster a therapeutic alliance. With the growing availability of digital health interventions for musculoskeletal conditions and the continuing high global prevalence and burden of chronic LBP, further research into the design, content and delivery of digital health technologies for low back pain is needed to optimise its acceptance by, and relevance for, individuals at risk of ongoing disability. The findings presented in this study provide important implications for clinical practice and future research in the management of chronic LBP.

## Supplementary Information

**Additional file 1.** Consolidated Criteria for Reporting Qualitative Research (COREQ) 32-item checklist.

## Data Availability

Release of participant data, including anonymised interviews is not possible due to the ethics committee requirements.
