# Supplementary material for: A process evaluation of the Mind Your Back trial examining psychologically informed physical treatments for chronic low back pain
Source: Chiropr Man Therap. 2021 Aug 17;29:32. doi: 10.1186/s12998-021-00389-y (PMC8369773; doi:10.1186/s12998-021-00389-y)
Supplement: Supplementary file 1 — Additional file 1. Consolidated Criteria for Reporting Qualitative Research (COREQ) 32-item checklist. [file 12998_2021_389_MOESM1_ESM.docx]

**Additional file 1:** Consolidated Criteria for Reporting Qualitative Research (COREQ): 32-item checklist.

| No Item | Guide questions/description | Information |
| --- | --- | --- |
| **Domain 1: Research team and reflexivity** |  |  |
| **Personal Characteristics** |  |  |
| 1. Interviewer/facilitator | Which author/s conducted the interview or focus group? | The lead researcher, MJP (male/ chiropractor/ researcher) PhD in health sciences research and clinical practice. MJP undertook this process evaluation as part of his PhD studies which was chiefly supervised by MM (male/ full-time researcher academic) PhD and auxiliary supervisor GS (female/ full-time qualitative researcher academic) PhD. Research team included GS who has extensive experience in social science research and is a specialist in qualitative research, and MM who has extensive health science and academic research experience. |
| 2. Credentials | What were the researcher’s credentials? E.g., PhD, MD | PhD candidate, BSc, MChiro |
| 3. Occupation | What was their occupation at the time of the study? | Chiropractor and PhD candidate |
| 4. Gender | Was the researcher male or female? | Male |
| 5. Experience and training | What experience or training did the researcher have? | The interviewer had limited experience in designing and conducting qualitative research prior to this study. However, GS, who oversaw every aspect of the qualitative process upheld rigour throughout the study and has extensive experience in qualitative research. Reflexivity and rigour was jointly upheld by MM who oversaw the project throughout the entire process and has extensive experience in academic research. |
| **Relationship with participants** |  |  |
| 6. Relationship established | Was a relationship established prior to study commencement? | The interviewer was involved in the recruitment and screening of many participants. However, participants in this study were not treated or had a therapeutic relationship with MJP which eliminated potential ethical, privacy issues. |
| 7. Participant knowledge of the  interviewer | What did the participants know about the researcher? E.g., personal goals, reasons for doing the  research | A basic knowledge of the interviewer was known to the interviewees. Primarily that MJP was the lead researcher for this study and that it was part of his PhD studies. |
| 8. Interviewer characteristics | What characteristics were reported about the interviewer/facilitator? E.g., bias, assumptions,  reasons and interests in the research topic | The interviews were conducted by the lead researcher (MJP) and directed by a topic discussion guide. MJP conducted this study as part of his PhD studies and was involved in the design and operational processes of the preceding clinical trial from where these participants were selected. Furthermore, MJP was involved in the formation of codes/ themes/ quotes that went towards the analysis and interpretation of this study. However, participants who took part in this qualitative study were not treated or had a therapeutic alliance with MJP at any point prior to the telephone interviews. |
| **Domain 2: study design** |  |  |
| **Theoretical framework** |  |  |
| 9. Methodological orientation and  Theory | What methodological orientation was stated to underpin the study? E.g., grounded theory,  discourse analysis, ethnography, phenomenology, content analysis | This study used a qualitative process evaluation methodology with a thematic analysis to uncover underling common themes experienced among participants. |
| **Participant selection** |  |  |
| 10. Sampling | How were participants selected? E.g., purposive, convenience, consecutive, snowball | Participants that had been treated by the interviewer in a professional capacity during the Mind Your Back trial were not invited for interview. Therefore, of the 108 trial participants, 61 were invited to take part in the process evaluation, of which a total of thirty-two participants agreed to be interviewed. |
| 11. Method of approach | How were participants approached? E.g., face-to-face, telephone, mail, email | Participants were contacted by telephone and introduced to the upcoming interview. Participants were informed of the general aims, time commitments and the nature of the questions that may be asked. If the participant verbally expressed interest over this telephone conversation, a Participant Information Sheet and consent form was emailed for consideration. In addition to written consent, verbal consent was acquired again at the time of the telephone interview.  Participants that had been treated by the interviewer in a professional capacity during the Mind Your Back trial were not invited for interview. |
| 12. Sample size | How many participants were in the study? | Of the 108 trial participants, 61 were invited to take part in the process evaluation, of which a total of thirty-two participants agreed to be interviewed. Twenty-five participants took part in the study. |
| 13. Non-participation | How many people refused to participate or dropped out? Reasons?  Setting | All invited participants agreed to participate in the telephone interviews. |
| 14. Setting of data collection | Where was the data collected? E.g., home, clinic, workplace | Telephone interviews were conducted privately at a mutually convenient time. In cases where the participant was not in a private location (e.g., they were at home with another person), they were given the option to reschedule the phone call to another time. |
| 15. Presence of non-participants | Was anyone else present besides the participants and researchers? | No |
| 16. Description of sample | What are the important characteristics of the sample? E.g., demographic data, date | The interviewed participants shared similar demographic characteristics to the 108 individuals in the Mind Your Back trial. That is, the interviewees were 12 women and 13 men, with an age range from 29 to 76, mean 53 (SD=13) who had lived with chronic low back pain for an average of 4.3 years. Furthermore, as in the Mind Your back trial which consisted of two evenly grouped intervention arms, interviews were conducted with fourteen individuals from group 1 (physical treatments only), and eleven from group 2 (combined MoodGYM and physical treatments). |
| **Data collection** |  |  |
| 17. Interview guide | Were questions, prompts, guides provided by the authors? Was it pilot tested? | The interview guide included open ended questions to enable discussion around aspects of the trial. |
| 18. Repeat interviews | Were repeat interviews carried out? If yes, how many? | No repeat interviews were conducted. |
| 19. Audio/visual recording | Did the research use audio or visual recording to collect the data? | Telephone interviews were digitally audio recorded and professionally transcribed verbatim. |
| 20. Field notes | Were field notes made during and/or after the interview or focus group? | Field notes were recorded during the telephone interview, taking particular attention of possible key points, emotional intonations/ emphasis that was embedded into the transcript for contextual detail. |
| 21. Duration | What was the duration of the interviews or focus group? | Interviews lasted between 20 to 45 minutes, mean 35 minutes. |
| 22. Data saturation | Was data saturation discussed? | Sample size was established by saturation of themes with ‘thick’ description of the data [1, 2]  which is a key criterion of the rigour of qualitative methods in determining sample size [3, 4]. |
| 23. Transcripts returned | Were transcripts returned to participants for comment and/or correction? | No this was not offered to participants. |
| **Domain 3: analysis and findings** |  |  |
| **Data analysis** |  |  |
| 24. Number of data coders | How many data coders coded the data? | There was one data coders: MJP, a PhD candidate with experience in health science research and clinical practice; The coding process was overlooked by Grace Spencer, who has extensive experience in social science research and is a specialist in qualitative research; and Martin Mackey, who has extensive health science and academic research experience. |
| 25. Description of the coding tree | Did authors provide a description of the coding tree? | Coding tables were assembled |
| 26. Derivation of themes | Were themes identified in advance or derived from the data? | Themes were derived from the data thematically without a predetermined theoretical framework. |
| 27. Software | What software, if applicable, was used to manage the data? | A basic word processing program (MS Word) was used for interview transcriptions, highlighting of major and minor themes, and generating a coding/ themes tree. |
| 28. Participant checking | Did participants provide feedback on the findings? | No this did not occur. |
| **Reporting** |  |  |
| 29. Quotations presented | Were participant quotations presented to illustrate the themes / findings? Was each  quotation identified? E.g., participant number | Participant quotations (as spoken) with gender and age were reported to illustrate themes and findings. |
| 30. Data and findings consistent | Was there consistency between the data presented and the findings? | Rigor maintained by three-person research team. |
| 31. Clarity of major themes | Were major themes clearly presented in the findings? | Two major themes are clearly presented in the findings |
| 32. Clarity of minor themes | Is there a description of diverse cases or discussion of minor themes? | No minor themes were identified in the data. |
|  |  |  |

NB: Based on the Consolidated Criteria for Reporting Qualitative Research (COREQ): 32-item checklist [5].

**References**

1. Holloway, I. and K. Galvin, *Qualitative research in nursing and healthcare*. 2016: John Wiley & Sons.

2. Denzin, N.K., *The research act: A theoretical introduction to sociological methods*. 2017: Routledge.

3. Morse, J.M., *The significance of saturation*. 1995, Sage Publications Sage CA: Thousand Oaks, CA.

4. Morse, J.M., *" Cherry picking": writing from thin data.* 2010.

5. Tong, A., P. Sainsbury, and J. Craig, *Consolidated criteria for reporting qualitative research (COREQ): a 32-item checklist for interviews and focus groups.* International Journal for Quality in Health Care, 2007. **19**(6): p. 349-57.
